# Perirenal fat thickness as a predictor of postoperative complications after laparoscopic distal gastrectomy for gastric cancer

**DOI:** 10.1002/bjs5.50338

**Published:** 2020-09-07

**Authors:** K. Eto, S. Ida, T. Ohashi, K. Kumagai, S. Nunobe, M. Ohashi, T. Sano, N. Hiki

**Affiliations:** ^1^ Department of Gastroenterological Surgery Cancer Institute Hospital Tokyo; ^2^ Department of Upper Gastrointestinal Surgery Kitasato University School of Medicine Sagamihara City Kanagawa Japan

## Abstract

**Background:**

Laparoscopic distal gastrectomy is used widely in surgery for gastric cancer. Excess visceral fat can limit the ability to dissect the suprapancreatic region, potentially increasing the risk of local complications, particularly pancreatic fistula. This study evaluated perirenal fat thickness as a surrogate for visceral fat to see whether this was related to complications after laparoscopic distal gastrectomy.

**Methods:**

Perirenal fat thickness was measured dorsal to the left kidney as an indicator of visceral fat in patients with gastric cancer who underwent laparoscopic distal gastrectomy. Patients were divided into two groups: those with and those without complications. The relationship between perirenal fat thickness and postoperative complications was evaluated.

**Results:**

The optimal cut‐off value for predicting morbidity using adipose tissue thickness was 10·7 mm; a distance equal to or greater than this was considered a positive perirenal fat thickness sign (PTS). A positive PTS showed a significant correlation with visceral fat area. Multivariable analysis found that a positive PTS was an independent risk factor for complications (hazard ratio 4·42, 95 per cent c.i. 2·31 to 8·86; *P* < 0·001).

**Conclusion:**

Perirenal fat thickness as an indicator of visceral fat was an independent predictor of postoperative complications after laparoscopic distal gastrectomy for gastric cancer.

## Graphical abstract

Perirenal fat thickness exhibited a significant correlation with visceral fat area. Furthermore, a positive perirenal fat thickness sign was associated with complications and was an independent risk factor after laparoscopic distal gastrectomy.

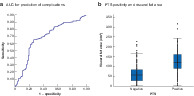

Novel indicator that merits evaluation in western patients

## Abstract (es)

**Antecedentes:**

La gastrectomía distal laparoscópica se utiliza ampliamente en la cirugía del cáncer gástrico. El exceso de grasa visceral puede limitar la capacidad para disecar la región suprapancreática, aumentando potencialmente riesgo de complicaciones locales, especialmente de fistula pancreática. El propósito de este estudio fue evaluar el grosor de la grasa perirrenal como marcador subrogado de grasa visceral para determinar si se relacionaba con complicaciones tras gastrectomía distal laparoscópica.

**Métodos:**

El grosor de la grasa perirrenal se midió a nivel dorsal del riñón izquierdo como indicador de grasa visceral en pacientes con cáncer gástrico sometidos a gastrectomía distal laparoscópica. Los pacientes fueron divididos en dos grupos: aquellos con y sin complicaciones. Se evaluó la relación entre grosor de la grasa perirrenal y las complicaciones postoperatorias.

**Resultados:**

El punto de corte óptimo para predecir la morbilidad utilizando el grosor del tejido adiposo fue de 10,7 mm, por lo que una distancia igual o mayor a este nivel fue considerado como signo positivo de engrosamiento de la grasa perirrenal (*peri‐renal fat thickness sign*, PTS). Un PTS positivo mostró una correlación significativa con el área de grasa visceral. Los análisis multivariables demostraban que un PTS positivo era un factor de riesgo independiente para complicaciones (razón de oportunidades, *odds ratio* 4,418; i.c. del 95% 2,307‐8,855; *P* < 0,001).

**Conclusión:**

El grosor de grasa perirrenal como indicador de la grasa visceral fue un predictor independiente de complicaciones postoperatorias tras una gastrectomía distal laparoscópica por cáncer gástrico.

## Introduction

Obesity is a technical limiting factor in abdominal surgery owing to the substantial operative difficulties caused by visceral fat and a narrow operating field. Many studies^1^, ^2^, ^3^, ^4^ have demonstrated that obesity and abundant visceral fat are associated with worse surgical outcomes, including increased duration of surgery, greater blood loss and more complications.

Laparoscopic distal gastrectomy has been adopted widely for surgery of gastric cancer^5^, ^6^, and clinical trials^7^, ^8^ have demonstrated that the outcomes of laparoscopic gastrectomy are similar or superior to those of open gastrectomy. The advantages of the laparoscopic approach include reduced postoperative pain, shorter hospital stay and quicker recovery^9^. However, a higher incidence of postoperative pancreatic fistula has been reported after laparoscopic distal gastrectomy compared with open gastrectomy^9^, ^10^. Lymph node dissection around the pancreas (stations 8, 9 and 11p) is important for curative resection of gastric cancer, but is made difficult by retroperitoneal, peripancreatic adipose tissue. This may account for the increased risk of pancreatic fistula.

**Fig. 1:**
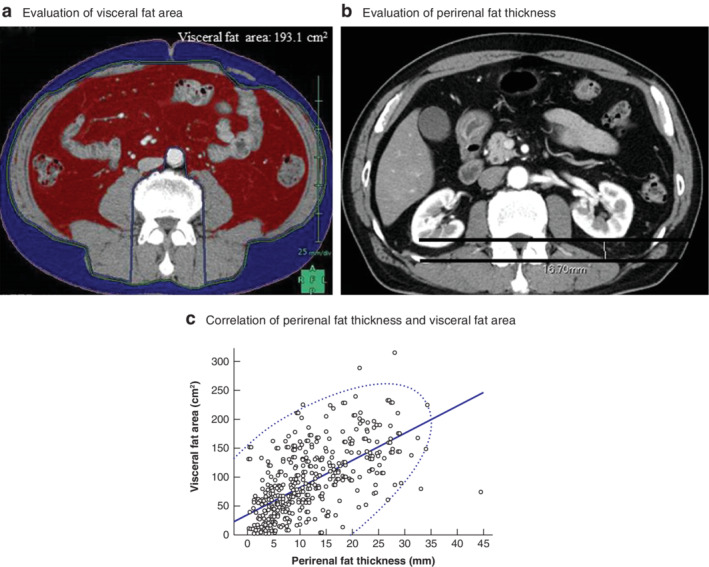
Evaluation of visceral fat area and perirenal fat thickness, and the correlation between them **a** Axial CT image at the level of the umbilicus, used to evaluate visceral fat area. **b** Axial CT image at the level of the distance from the anterior margin of the quadratus lumborum muscle to the dorsal margin of the left renal pole, used to evaluate the thickness of adipose tissue dorsal to the left kidney. **c** Correlation between adipose tissue thickness dorsal to the left kidney and visceral fat area. The curved line shows the probability ellipse (*R* ^2^ = 0·61, *P* < 0·001).

Perirenal fat, measured using CT images, has been reported^11^, ^12^, ^13^ as an easily reproducible, indirect measurement that correlates with visceral fat. These studies found an association between the volume of perirenal fat and postoperative complications after colorectal surgery, but this association has not been investigated in surgery for gastric cancer.

This study focused on the thickness of adipose tissue dorsal to the left kidney as an indicator of visceral fat. The aim of the study was to measure perirenal fat thickness and to investigate the relationship between this and postoperative complications after laparoscopic distal gastrectomy.

## Methods

Data collection and analysis were approved by the institutional scientific review board of the Cancer Institute Hospital (number 0171).

Patients with gastric cancer of clinical stage cT1 N0, cT2 N0 or cT1 N1 were offered laparoscopic distal gastrectomy in accordance with the Japanese Gastric Cancer Association treatment guidelines^14^. Clinical staging consisted of upper endoscopy, endoscopic ultrasonography, thoracoabdominal CT and barium radiography. D1+ lymph node dissection was performed for patients with cT1 N0 disease, and D2 dissection for the other stages.

**Fig. 2:**
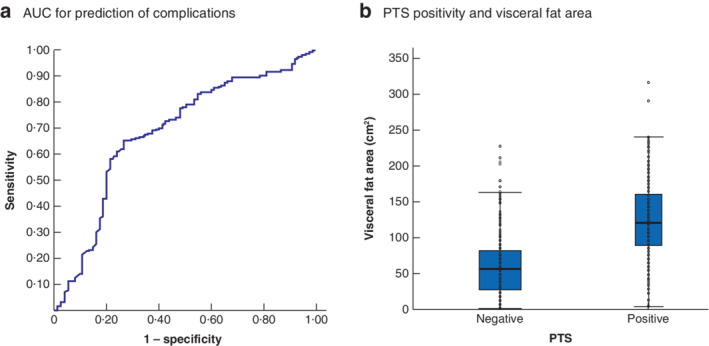
Prediction of postoperative complications using preoperative perirenal fat thickness, and relationship between perirenal fat thickness positivity and visceral fat area **a** Receiver operating characteristic (ROC) curve for predicting complications rated as grade II or higher according to the Clavien–Dindo classification using preoperative measurement of the thickness of adipose tissue dorsal to the left kidney (area under the ROC curve (AUC) = 0·71). **b** Visceral fat area in patients with a positive or negative perirenal fat thickness sign (PTS). Individual patient data are shown, and box plots indicate median values, interquartile ranges and ranges, denoted by horizontal bars, boxes and error bars respectively (*P* < 0·001, Wilcoxon signed‐rank test).

Consecutive patients with gastric cancer who underwent laparoscopic distal gastrectomy at the Cancer Institute Hospital of the Japanese Foundation for Cancer Research, Tokyo, Japan, from January 2013 to December 2015 were included in the analysis. Informed consent was obtained from all participants included in the study. Clinical and demographic information was obtained from hospital and surgical records, including patient age, sex, BMI, ASA physical status, blood test results, duration of surgery, recorded blood loss, reconstruction method and postoperative complications.

The severity of postoperative complications was evaluated according to the Clavien–Dindo classification system^15^. Postoperative complications were included if they were Clavien–Dindo grade II or higher, and categorized to identify anastomotic leakage, pancreatic fistula, intra‐abdominal infection, surgical‐site infection and pneumonia. Patients were divided into two groups: those with complications and those without.

### Measurement of visceral fat area

The visceral fat area was evaluated using preoperative CT images created at the level of the umbilicus. A three‐dimensional image analysis system (Volume Analyzer SYNAPSE VINCENT; Fujifilm Medical, Tokyo, Japan) was used to measure pixels using a window width of −30 to 150 Hounsfield units to delineate the muscle and fat compartments and calculate the cross‐sectional area of each in square centimetres (*Fig*. *1a*).

### Measurement of adipose tissue thickness dorsal to the kidney

The distance from the anterior margin of the quadratus lumborum muscle to the dorsal margin of the left renal pole at the level of the point of exit of the renal vein was measured on a transverse section CT image (*Fig*. *1b*). This distance was defined as the thickness of the adipose tissue dorsal to the left kidney (perirenal fat thickness).

### Statistical analysis

The sensitivity and specificity of the nominated variables to predict postoperative complications were assessed using receiver operating characteristic (ROC) curve analysis. Goodness‐of‐fit was assessed by calculating the area under the curve (AUC), and the optimal cut‐off value was determined using the Youden index.

Clinicopathological characteristics and laboratory data in the two groups were compared with the χ^2^ test for categorical variables and Wilcoxon signed‐rank test for continuous variables. *P* < 0·050 was considered statistically significant. A Cox proportional hazards model was used to assess the effects of co‐variables in both univariable and multivariable analyses. Multivariable analysis was performed using factors from the univariable analysis with *P* < 0·050. All tests were analysed using JMP® software (SAS Institute, Cary, North Carolina, USA).

## Results

A total of 476 patients were included with a mean age of 64·5 years, of whom 310 were men.

### Correlation between visceral fat and perirenal fat thickness

There was a moderate correlation coefficient of 0·62 between the visceral fat area and perirenal fat thickness (*P* < 0·001) (*Fig*. *1c*). The AUC value, indicating the power to predict postoperative complications, was 0·71 for preoperative perirenal fat thickness (*Fig*. *2a*), and the optimal cut‐off value for predicting morbidity using the perirenal fat thickness was 10·7 mm. Adipose tissue thickness of 10·7 mm or more was defined as a positive perirenal fat thickness sign (PTS). PTS‐positive patients had a significantly greater visceral fat area than PTS‐negative patients (*Fig*. *2b*).

A total of 70 patients (14·7 per cent) developed complications. In this group a significantly greater proportion of patients were men, and patients had a higher preoperative BMI, a greater distance from the anterior margin of the quadratus lumborum muscle to the dorsal margin of the left renal pole, and a higher rate of PTS positivity than patients with no complications. Patients with complications also experienced significantly longer operating times and greater blood loss (*Table* *1*).

**Table 1:** Association between perioperative patient characteristics and postoperative morbidity in the two groups

	Complications (*n* = 70)	No complications (*n* = 406)	*P* §
**Age (years)** *	69·5 (42–91)	67 (25–98)	0·055¶
**Sex ratio (M** : **F)**	55 : 15	255 : 151	< 0·001
**BMI (kg/m** ^**2**^ **)** *	23·5 (16·1–31·0)	22·4 (14·5–39·1)	< 0·001¶
**ASA grade before anaesthesia**			0·074
I	33 (47)	247 (60·8)	
II	35 (50)	155 (38·2)	
III	2 (3)	4 (1·0)	
**Gastric tumour location**			0·760
Upper	6 (9)	42 (10·3)	
Middle	38 (54)	230 (56·7)	
Low	26 (37)	134 (33·0)	
**Clinical stage** **‡**			0·492
IA	61 (87)	341 (84·0)	
IB	9 (13)	65 (16·0)	
**Thickness of adipose tissue dorsal to left kidney (mm)** *	13·8 (0·2–32·9)	8·3 (0·2–44·2)	< 0·001¶
**Positive PTS**	49 (70)	146 (36·0)	< 0·001
**Preoperative total protein (g/dl)** *	6·9 (5·5–7·8)	6·8 (5·5–8·1)	0·335¶
**Preoperative albumin (g/dl)** *	4·1 (3·1–4·7)	4·1 (2·8–4·9)	0·836¶
**Preoperative prealbumin (mg/dl)** *	26·7 (11·6–48·7)	27·6 (17·8–41·2)	0·129¶
**Preoperative haemoglobin (g/dl)** *	13·7 (8·4–16·1)	13·5 (8·9–16·7)	0·182¶
**Lymph node dissection**			0·548
D1+	58 (83)	324 (79·8)	
D2	12 (17)	82 (20·2)	
**Type of reconstruction**			0·223
Billroth I	29 (41)	137 (33·7)	
Roux‐en‐Y	41 (59)	269 (66·3)	
**Duration of surgery (min)** *	292 (175–432)	280 (156–489)	0·033¶
**Blood loss (ml)** *	35 (3–500)	10 (0–560)	0·012¶
**Postoperative hospital stay (days)** **†**	15 (8–71)	9 (6–36)	< 0·001¶

Values in parentheses are percentages unless indicated otherwise; values are

* median (range) and

† mean (range).

‡ According to the seventh edition of the IUCC TNM classification system. PTS, perirenal fat thickness sign.

§ χ^2^ test, except

¶ Wilcoxon signed‐rank test.

A total of 195 patients (41·0 per cent) were included in the PTS‐positive group. This group contained a significantly higher proportion of men, and patients had a greater preoperative BMI. They were more likely to have undergone Roux‐en‐Y reconstruction, had longer operating times, greater volumes of blood loss, and experienced higher complication rates leading to longer postoperative hospital stays than the other patients (*Table* *2*).

**Table 2:** Association between perioperative patient characteristics and perirenal fat thickness sign in the two groups

	PTS‐positive group (*n* = 195)	PTS‐negative group (*n* = 281)	*P* §
**Age (years)** *	68 (35–91)	66 (25–98)	0·120¶
**Sex ratio (M** : **F)**	179 : 16	131 : 150	< 0·001
**BMI (kg/m** ^**2**^ **)** *	24·2 (18·1–39·1)	21·4 (14·5–30·8)	< 0·001¶
**ASA grade before anaesthesia**		*n* = 280	0·350
I	110 (56·4)	169 (60·4)	
II	81 (41·5)	109 (38·9)	
III	4 (2·1)	2 (0·7)	
**Gastric tumour location**			0·137
Upper	26 (13·3)	22 (7·8)	
Middle	108 (55·4)	160 (56·9)	
Low	61 (31·3)	99 (35·2)	
**Clinical stage** **‡**			0·491
IA	162 (83·1)	240 (85·4)	
IB	33 (16·9)	41 (14·6)	
**Preoperative total protein (g/dl)** *	6·9 (5·5–7·8)	6·8 (5·5–8·1)	0·183¶
**Preoperative albumin (g/dl)** *	4·1 (3·1–4·8)	4·1 (2·8–4·9)	0·732¶
**Preoperative prealbumin (mg/dl)** *	27·7 (16·3–48·7)	26·1 (11·6–42·4)	< 0·001¶
**Preoperative haemoglobin (g/dl)** *	13·9 (8·4–16·7)	13·1 (9·6–15·9)	< 0·001¶
**Lymph node dissection**			0·908
D1+	156 (80·0)	226 (80·4)	
D2	39 (20·0)	55 (19·6)	
**Type of reconstruction**			0·019
Billroth I	56 (28·7)	110 (39·1)	
Roux‐en‐ Y	139 (71·3)	171 (60·9)	
**Duration of surgery (min)** *	298 (160–489)	273 (156–470)	< 0·001¶
**Blood loss (ml)** *	30 (0–560)	15 (0–470)	< 0·001¶
**Postoperative complications**	49 (25·1)	21 (7·5)	< 0·001
**Postoperative inflammatory complication**	39 (20·0)	6 (2·1)	< 0·001
**Postoperative hospital stay (days)** **†**	10 (7–71)	9 (6–56)	0·004¶

Values in parentheses are percentages unless indicated otherwise; values are

* median (range) and

† mean (range).

‡ According to the seventh edition of the IUCC TNM classification system. PTS, perirenal fat thickness sign.

§ χ^2^ test, except

¶ Wilcoxon signed‐rank test.

**Table 3:** Univariable and multivariable analysis of factors affecting postoperative morbidity following laparoscopic distal gastrectomy

	Univariable analysis		Multivariable analysis	
	Hazard ratio	*P*	Hazard ratio	*P*
**Age (years)**				
< 75	1·00 (reference)			
≥ 75	1·63 (0·95, 2·76)	0·077		
**Sex**				
F	1·00 (reference)		1·00 (reference)	
M	2·20 (1·25, 4·09)	0·006	1·36 (0·63, 2·91)	0·426
**BMI (kg/m** ^**2**^ **)**				
< 25	1·00 (reference)		1·00 (reference)	
≥ 25	2·59 (1·51, 4·39)	< 0·001	1·42 (0·79, 2·53)	0·236
**ASA grade before anaesthesia**				
I	1·00 (reference)		1·00 (reference)	
II–III	1·67 (1·02, 2·74)	0·043	1·70 (0·98, 2·90)	0·058
**Gastric tumour location**				
Upper	1·00 (reference)			
Middle/low	0·93 (0·45, 2·29)	0·856		
**Clinical tumour depth** *				
cT1	1·00 (reference)			
cT2	1·29 (0·63, 2·90)	0·495		
**Previous history of surgery**				
No	1·00 (reference)			
Yes	0·91 (0·53, 1·63)	0·753		
**Clinical stage** *				
IA	1·00 (reference)			
IB	1·04 (0·50, 1·98)	0·906		
**Preoperative albumin (g/dl)**				
< 4·1	1·00 (reference)			
≥ 4·1	0·86 (0·52, 1·42)	0·570		
**Preoperative prealbumin (mg/dl)**				
< 27·0	1·00 (reference)			
≥ 27·0	0·62 (0·37, 1·05)	0·078		
**Preoperative total protein (g/dl)**				
< 6·9	1·00 (reference)			
≥ 6·9	0·69 (0·42, 1·15)	0·158		
**Preoperative haemoglobin (g/dl)**				
< 13·5	1·00 (reference)		1·00 (reference)	
≥ 13·5	0·60 (0·36, 0·99)	0·044	0·80 (0·45, 1·41)	0·440
**Duration of surgery (min)**				
< 280	1·00 (reference)			
≥ 280	1·39 (0·84, 2·30)	0·191		
**Blood loss (ml)**				
< 20	1·00 (reference)		1·00 (reference)	
≥ 20	2·46 (1·48, 4·16)	< 0·001	1·72 (0·97, 2·53)	0·059
**Positive PTS**				
No	1·00 (reference)		1·00 (reference)	
Yes	5·13 (3·00, 9·08)	< 0·001	4·42 (2·31, 8·86)	< 0·001
**Lymph node dissection**				
D1+	1·00 (reference)			
D2	1·09 (0·59, 2·11)	0·800		
**Type of reconstruction**				
Billroth I	1·00 (reference)			
Roux‐en‐Y	0·77 (0·47, 1·29)	0·304		

Values in parentheses are 95 per cent confidence intervals.

* According to the seventh edition of the IUCC TNM classification system. PTS, perirenal fat thickness sign.

### Risk factors for postoperative complications

In the multivariable analysis, only PTS positivity was identified as an independent predictive factor for postoperative complications (hazard ratio 4·42, 95 per cent c.i. 2·31 to 8·86; *P* < 0·001) (*Table* *3*). Positive PTS was also the only independent predictive factor for postoperative inflammatory complications (hazard ratio 7·75, 3·04 to 23·27; *P* < 0·001) and a predictor for pancreatic fistula and anastomotic leakage (*Tables* *S1* and *S2*, supporting information).

## Discussion

This study has demonstrated that perirenal fat thickness is a simple and useful predictor for the development of complications after laparoscopic distal gastrectomy in patients with gastric cancer. A positive PTS can be used to identify patients at increased risk of developing complications after laparoscopic distal gastrectomy, consider modifications in management, and influence the consent process.

Despite advances in laparoscopic gastric cancer surgery and perioperative care that have resulted in reduced hospital mortality, postoperative complications remain a significant problem affecting 20–46 per cent of patients^16^, ^17^, ^18^. Other studies^19^, ^20^ have also suggested that the occurrence of postoperative complications, particularly inflammatory complications, can impact negatively on the long‐term prognosis of these patients. Identifying patients at high risk of developing complications is important to introduce strategies that might prevent their occurrence.

BMI is used widely as an indicator of obesity. Higher BMI has been associated with increased complications after gastrectomy, including laparoscopic distal gastrectomy, in several studies^2^, ^4^, ^16^, ^21^, although not all^22^. These studies did not consider the relationship between BMI, visceral fat and postoperative complications. As BMI is calculated using bodyweight and height, it includes both visceral and subcutaneous fat, but does not determine the relative proportions of each component. Precise measurement of the entire visceral fat volume is complex, requires specialized software, and seems unsuitable as a clinical tool. The present study focused on a novel indicator, perirenal fat thickness, to determine whether this might be surrogate marker of visceral fat. The study confirmed that the thickness of perirenal fat correlated with visceral fat volume and predicted a substantially increased risk of postoperative complications after laparoscopic distal gastrectomy. The simplicity with which perirenal fat thickness can be measured makes it a clinically valuable tool for evaluating visceral fat obesity as a means of identifying patients at increased risk of developing postoperative complications. Local complications, including pancreatic fistula and anastomotic leakage, are thought to be more likely when dissection is made more difficult by abundant visceral fat and a narrow operating field, and the present study appeared to support this.

Previous studies have reported various systems for predicting surgical risk. The POSSUM score^23^, modified POSSUM^24^, and Estimation of Physiological Ability and Surgical Stress (E‐PASS) scoring system^25^ have been reported to provide reliable predictive scores for mortality and morbidity. These are scoring systems for predicting total morbidity, but they use both preoperative and intraoperative factors to predict morbidity and mortality. The strength of the risk prediction score proposed in the present study is that it relies solely on preoperative risk factors.

Preoperative measurement of perirenal fat thickness can be added to the clinical decision‐making process when estimating the risk of adverse outcomes for patients with gastric cancer. This could assist during both patient counselling and the informed consent process. The identification of high‐risk patients may allow perioperative care to be tailored to their needs and improve short‐term outcomes, and raises the possibility that the risk of complications in PTS‐positive patients might be modified by weight loss before surgery.

There are several limitations to this study. It was a cohort study conducted at a single institution, subject to the biases inherent in this approach. A prospective multi‐institutional study is needed to validate the present findings, and interobserver reliability for these measurements should be demonstrated.

## Disclosure

The authors declare no conflict of interest.

## Supporting information


**Table S1** Univariable and multivariable analysis of factors affecting postoperative inflammatory complications following laparoscopic distal gastrectomy
**Table S2** Association between postoperative complications after laparoscopic distal gastrectomy for gastric cancer and the perirenal fat thickness sign
