# Supplementary material for: Perirenal fat thickness as a predictor of postoperative complications after laparoscopic distal gastrectomy for gastric cancer
Source: BJS Open. 2020 Sep 7;4(5):865–72. doi: 10.1002/bjs5.50338 (PMC7528519; doi:10.1002/bjs5.50338)
Supplement: Supplementary file 1 — Table S1 Univariable and multivariable analysis of factors affecting postoperative inflammatory complications following laparoscopic distal gastrectomy Table S2 Association between postoperative complications after laparoscopic distal gastrectomy for gastric cancer and the perirenal fat thickness sign [file BJS5-4-865-s001.docx]

**BJS5_50338**

**Perirenal fat thickness as a predictor of postoperative complications after laparoscopic distal gastrectomy for gastric cancer**

**K. Eto, S. Ida, T. Ohashi, K. Kumagai, S. Nunobe, M. Ohashi, T. Sano and N. Hiki**

**Table S1** Univariable and multivariable analysis of factors affecting postoperative inflammatory complications following laparoscopic distal gastrectomy

| Factors | Univariate analysis | | | Multivariate analysis | | |
| --- | --- | --- | --- | --- | --- | --- |
|  | HR | 95% CI | P-value | HR | 95% CI | P-value |
| Age |  |  |  |  |  |  |
| <75 years | 1.000 |  |  |  |  |  |
| ≧75 years | 1.185 | 0.588-2.600 | 0.647 |  |  |  |
| Gender |  |  |  |  |  |  |
| Female | 1.000 |  |  | 1.000 |  |  |
| Male | 3.348 | 1.708-10.27 | <0.001 | 1.388 | 0.439-4.596 | 0.561 |
| Body mass index |  |  |  |  |  |  |
| <25 kg/m^2^ | 1.000 |  |  | 1.000 |  |  |
| ≧25 kg/m^2^ | 4.133 | 2.188-7.803 | <0.001 | 1.912 | 0.960-3.809 | 0.075 |
| Preoperative Anaesthesia Physical Status score |  |  |  |  |  |  |
| I | 1.000 |  |  |  |  |  |
| II/III | 1.544 | 0.833-2.871 | 0.167 |  |  |  |
| Tumour location |  |  |  |  |  |  |
| Upper | 1.000 |  |  |  |  |  |
| Middle/Low | 0.899 | 0.331-2.672 | 0.814 |  |  |  |
| Clinical tumour depth ^a^ |  |  |  |  |  |  |
| cT1 | 1.000 |  |  |  |  |  |
| cT2 | 1.200 | 0.490-3.021 | 0.814 |  |  |  |
| Preoperative history |  |  |  |  |  |  |
| - | 1.000 |  |  |  |  |  |
| + | 0.920 | 0.470-1.912 | 0.814 |  |  |  |
| Clinical stage ^a^ |  |  |  |  |  |  |
| cStage IA | 1.000 |  |  |  |  |  |
| cStage IB | 1.200 | 0.500-2.561 | 0.669 |  |  |  |
| Preoperative albumin |  |  |  |  |  |  |
| <4.1 g/dl | 1.000 |  |  |  |  |  |
| ≧4.1 g/dl | 0.957 | 0.564-1.948 | 0.889 |  |  |  |
| Preoperative prealbumin |  |  |  |  |  |  |
| <27.0 mg/dl | 1.000 |  |  |  |  |  |
| ≧27.0 mg/dl | 0.591 | 0.307-1.108 | 0.101 |  |  |  |
| Preoperative total protein |  |  |  |  |  |  |
| <6.9 g/dl | 1.000 |  |  |  |  |  |
| ≧6.9 g/dl | 0.872 | 0.468-1.641 | 0.667 |  |  |  |
| Preoperative haemoglobin |  |  |  |  |  |  |
| <13.5 | 1.000 |  |  | 1.000 |  |  |
| ≧13.5 | 0.449 | 0.232-0.841 | 0.012 | 0.782 | 0.630-2.659 | 0.500 |
| Operation time |  |  |  |  |  |  |
| <280 min | 1.000 |  |  | 1.000 |  |  |
| ≧280 min | 2.104 | 1.117-4.122 | 0.021 | 1.203 | 0.590-2.518 | 0.613 |
| Blood loss |  |  |  |  |  |  |
| <20 ml | 1.000 |  |  | 1.000 |  |  |
| ≧20 ml | 6.092 | 2.912-14.37 | <0.001 | 3.603 | 1.574-9.124 | 0.0019 |
| PTS |  |  |  |  |  |  |
| - | 1.000 |  |  | 1.000 |  |  |
| + | 11.46 | 5.010-30.67 | <0.001 | 7.747 | 3.041-23.27 | <0.001 |
| Lymph node dissection |  |  |  |  |  |  |
| D1+ | 1.000 |  |  |  |  |  |
| D2 | 1.018 | 0.446-2.108 | 0.964 |  |  |  |
| Reconstruction |  |  |  |  |  |  |
| Billroth I | 1.000 |  |  |  |  |  |
| Roux en Y | 0.784 | 0.421-1.492 | 0.452 |  |  |  |

^a^ According to the 7th edition of the International Union Against Cancer tumour, node, metastasis classification system

**Table S2** Association between postoperative complications after laparoscopic distal gastrectomy for gastric cancer and the perirenal fat thickness sign

| Factors | PTS-positive group | PTS-negative group | P-value |
| --- | --- | --- | --- |
|  | (N=195) | (N=281) |  |
| Intraabdominal abscess, n (%) |  |  | <0.001 |
| + | 22 (11.28%) | 5 (1.779%) |  |
| Pancreatic fistula, n (%) |  |  | 0.0022 |
| + | 10 (5.13%) | 2 (0.712%) |  |
| Anastomotic leakage, n (%) |  |  | 0.023 |
| + | 7 (3.59%) | 2 (0.712%) |  |
| Pneumonia, n (%) |  |  | 0.073 |
| + | 4 (2.05%) | 0 (0%) |  |
| Postoperative bleeding, n (%) |  |  | 0.603 |
| + | 4 (2.05%) | 4 (1.42%) |  |
| Bowel obstruction, n (%) |  |  | 0.785 |
| + | 1 (0.513%) | 2 (0.712%) |  |
